# Assessing System Thinking in Senior Pharmacy Students Using the Innovative “Horror Room” Simulation Setting: A Cross-Sectional Survey of a Non-Technical Skill

**DOI:** 10.3390/healthcare11010066

**Published:** 2022-12-26

**Authors:** Lobna A. Aljuffali, Haya M. Almalag, Lamya Alnaim

**Affiliations:** Department of Clinical Pharmacy, College of Pharmacy, King Saud University, Riyadh 11149, Saudi Arabia

**Keywords:** horror room, system thinking, errors, patient safety, pharmacy education, non-technical skills

## Abstract

System thinking is an important competency for all healthcare professionals as it is a required skill to provide safe patient care. However, the literature does not describe how students gain such a skill or the manner in which it is assessed. **Purpose:** This study aimed to assess pharmacy students’ non-technical skills in the form of system thinking and error detection in a simulated setting. Results were correlated with the number of errors students were able to identify in a team-based simulation activity called the “horror room.” **Patients and methods:** A cross-sectional survey was administered after completion of the “horror room” simulation activity to identify elements of system thinking and error detection. Survey respondents were senior students enrolled in a patient safety course. System thinking elements identified in the survey were then linked to the number of errors reported. **Results:** Sixty-six students participated in the activity. Their mean grade point average (GPA) was 4.72 (standard deviation (SD) 0.22), and the mean number of errors detected was 8 (SD 2). The average total system thinking score (STS) was 68 (SD 8.4). There was no association between the number of errors detected and STS; however, a positive association was found between GPA and STS (Spearman’s correlation coefficient = 0.27, *p* = 0.030). The most common type of error detected was a medication safety error (100%). **Conclusions:** High STS showed that teaching theory is important for students to learn concepts; however, knowing the ideas associated with system thinking does not necessarily translate into practice, as evidenced by the low number of errors students were able to detect.

## 1. Introduction

The healthcare system is a complex system in which simple tasks such as dispensing medication to a patient entail a multistep process involving computers, physicians, pharmacists, and nurses, where each professional performs a task that is integral to another. Failures and mishaps can occur at any step, making it hard to identify the exact cause contributing to the resultant harm [1,2,3]. A complex system involves numerous interacting parts and comprises a structure and behavioral patterns that are difficult to understand and predict [4]. It has been demonstrated that the complexity of factors in healthcare is an important consideration for patient safety [1]. Hence, a holistic approach to identifying the factors involved in a system is crucial to develop an understanding of the interconnection among factors as well as how interactions occur and relate to one another and ultimately how patient safety may be impacted. 

System thinking is considered a holistic approach as it helps with understanding processes [5], predicting system behavior, and improving system design [6,7,8]. The term is defined as “the ability to recognize, understand, and synthesize the interactions and interdependencies in a set of components designed for a specific purpose.” This includes the ability to recognize patterns and repetitions in interactions and an understanding of how actions and components can reinforce or counteract each other [9,10].

Providing pharmaceutical care is an integral part of the pharmacist’s role in healthcare, where care is provided centered around the patient to improve their quality of life. System thinking is an approach that helps to address all patient needs in a systematic process to ensure the best care is delivered [11,12].

The system thinking approach can help students develop essential skills by identifying patterns, understanding problems’ root causes, analyzing contributory factors, and designing systematic improvement processes that are robust to healthcare professionals’ workload and stress. In a study assessing nurses’ perceptions of system thinking and safe nursing care, a positive correlation was found between system thinking and safe nursing care [10]. 

System thinking is a skill that enhances problem solving and clinical reasoning [13]. The Accreditation Council on Pharmacy Education (ACPE) [14] has identified important related skills in Standards 3 (Approach to Practice and Care) and 4 (Personal and Professional Development), including the non-technical skills of critical thinking, problem solving, leadership, and communication [15]. 

System thinking teaching requires didactic, experiential, and reflective learning, as it is a higher level of thinking and should be started from the pre-medical stage and continued through professional development. Different courses across curricula should include system thinking. A study identified courses such as quality improvement, inter-professional education (IPE), error mitigation, and advocacy as suitable to teach system thinking. In the healthcare curriculum, system thinking has been taught in an unplanned and unsystematic manner [15]. Further, the literature does not fully elucidate the method through which students gain such a skill and how it is assessed. 

System thinking is part of the patient safety course in the Pharm D program offered at King Saud University, College of Pharmacy [16]. The course is based on the World Health Organization’s (WHO) *Patient Safety Curriculum Guide: Multi-Professional Edition* [17]. Regular assessment tools such as multiple questions and case studies are insufficient to assess this high-level skill as it needs to be evaluated in a real environment in the context of a team where there is a problem to be solved. This study aimed to assess pharmacy students’ system thinking perceptions and the correlation with the number of errors they were able to identify in a team-based simulation activity called the “horror room”.

## 2. Material and Methods

The present study entailed a cross-sectional observational activity that was part of a planned simulation activity in a patient safety course involving the previous year’s (2020) Pharm D students. The activity occurred after students completed 6 weeks of didactic lectures on patient safety topics (including one lecture on system thinking) with a focus on the importance of non-technical skills (NTS). The 6-year ACPE-accredited Pharm D program at King Saud University consists of didactic and practical courses during the first 5 years and an internship in the final year. The courses are offered on two campuses, one for men and the other for women [16].

### 2.1. Study Design and Setting 

The study comprised Phase I, which entailed preparing for the simulation activity, and Phase II, which entailed a working group’s cross-sectional evaluation of NTS among the College of Pharmacy students. King Saud University, College of Pharmacy in Riyadh, Saudi Arabia, has established two standard simulation rooms at two campuses [16]. The rooms simulate a hospital inpatient room setting with simulated patients full of hidden errors, either by commission or omission (e.g., patient given Ampicillin despite documented penicillin allergy, wrong patient name on one page of the medication record, instructions to give oral instead of IV despite ‘nothing by mouth’ sign on the bed, patient with a fall risk sign and side rails down, etc.), Appendix 1. The room was named “the room of horrors” [17,18]. The study was conducted on the women’s campus only.

The study was conducted according to the guidelines of the Declaration of Helsinki and approved by the Institutional Review Board Committee of King Saud University Medical City (protocol code E-21-5892 and 27 April 2021).

### 2.2. Participants in NTS Assessment

Participants were women Pharm D students in their final year of the Pharm D program registered in the patient safety course in 2020, which is a required course in the PharmD program. 

### 2.3. Phase I—Simulation Activity Preparation and Operationalization

#### 2.3.1. Personnel Involved

A working group was established comprising two faculty members and three interning Pharm D students. The group was responsible for preparing the case, the equipment, and the simulated inpatient room, in addition to overseeing the organized activity and conducting the NTS evaluation.

#### 2.3.2. Simulation and Equipment 

The working group prepared the simulation case scenario based on the International Patient Safety Goals (IPSG) and the WHO patient safety curriculum taught to students during the course [17,19]. The setting was designed based on Farnan et al. [18]. The students were required to draw upon their background knowledge and common sense to conduct observations of the five principles of medication prescription/dispensation, namely selecting the appropriate antibiotics based on patient diagnoses, implementing basic infection control measures, accounting for allergies and drug–food interactions, identifying patients with a high risk of falling, and ensuring proper medication storage. The case was designed and piloted on two students. Five patient safety issues and 23 errors were present in the activity setting (see Appendix 1 for details on included errors) [19].

#### 2.3.3. The Activity

After completing 6 weeks of didactic lectures, the students were assigned to groups of three, and each group was assigned a number between 1 and 26. All students provided their grade point average (GPA), which in King Saud University the GPA is out of 5. Prior to entering the patient room, students received a brief introduction to the activity and a clipboard with a list. Each group spent 5 min in the patient room. They inspected the patient's file and the settings in the room to identify factors that could lead to harm to the patient. They also identified and recorded errors. A faculty member and an intern were present in each room. At the end of each 5-min session, each student group turned in their clipboard, and the intern assisted the faculty member with re-arranging and re-organizing. The activity carried a weight of 2% of the total course grade 2 out of 100.

### 2.4. Phase II—Post-Activity NTS Assessment

#### 2.4.1. Assessment Tools and Measurements to Detect NTS

After completing the activity, the student’s grasp of system thinking was captured using the system thinking score (STS) [20]. The STS is a 20-item user-friendly instrument that has been proven to be valid and reliable. The STS measures the system thinking construct of system interdependencies in the context of quality improvement. It uses a 5-point Likert scale, where 0 = *Never*, 1 = *Seldom*, 2 = *Sometimes*, 3 = *Often*, and 4 = *Most of the time*. The total score is computed by summing the responses for each item. Scores can range from 0 to 80. Applying the STS is a viable option for assessing system thinking in both clinical and educational settings. Permission to use the STS was obtained from its primary author. The number of errors identified and each student’s STS score were recorded.

#### 2.4.2. Analysis

Data were coded and input to Microsoft Excel and the IBM Statistical Package for Social Sciences (SPSS) version 27 (Armonk, USA). The mean and standard deviation for the continuous variables and the number or percent of the categorical variables were reported whenever appropriate. The relationships between GPA and STS and between the number of errors identified and STS were explored using Spearman’s correlation coefficient. *p*-values less than 0.05 were considered significant. 

## 3. Results

A total of 76 students in 26 groups performed the activity, and 66 students completed the STS, response rate of 87%. The students had a mean (SD) GPA of 4.72 (0.22). In their groups, they found a mean (SD) of 8 errors. Additionally, the mean (SD) total STS was 62.8 (8.4). The most common types of errors identified were medication-related issues (*n* = 66, 100%), followed by errors related to hospital-acquired infections (*n* = 60, 91%) and nothing by mouth (*n* = 60, 91%), errors related to fall risk (*n* = 33, 50%), and finally, deep venous thrombosis prophylaxis (*n* = 22, 33%). No association between error type and STS or number of errors identified was found. 

**Figure 1:**
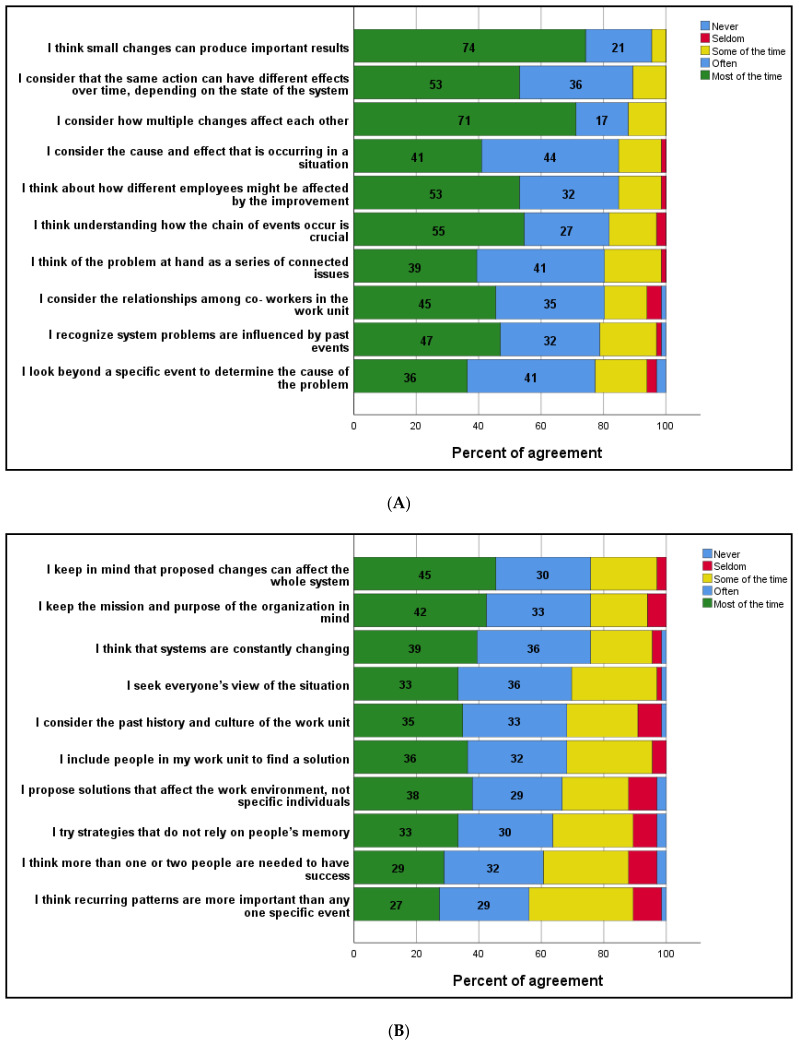
Students’ level of agreement with system thinking survey items. (**A**), Part 1. (**B**), Part 2.

Regarding the following system thinking statements, most students responded “agree” or “most of the time” (percentage of total respondents indicated in parentheses):

“I think small changes can produce important results” (95%), Figure 1A; “I consider that the same action can have different effects over time depending on the state of the system” (89%), Figure 1A; “I consider how multiple changes affect each other” (88%), Figure 1A; “I consider the cause and effect that is occurring in a situation” (85%), Figure 1A; “I think about how different employees might be affected by the improvement” (85%), Figure 1A; “I think understanding how the chain of events occur is crucial” (82%), Figure 1A; “I think of the problem at hand as a series of connected issues” (80%), Figure 1A; “I consider the relationship among co-workers in the work unit” (80%), Figure 1A; “I keep in mind that proposed changes can affect the whole system” (75%), Figure 1B. The data for system thinking statements and students’ degree of agreement with each statement are available in Figure 1A,B.

**Figure 2:**
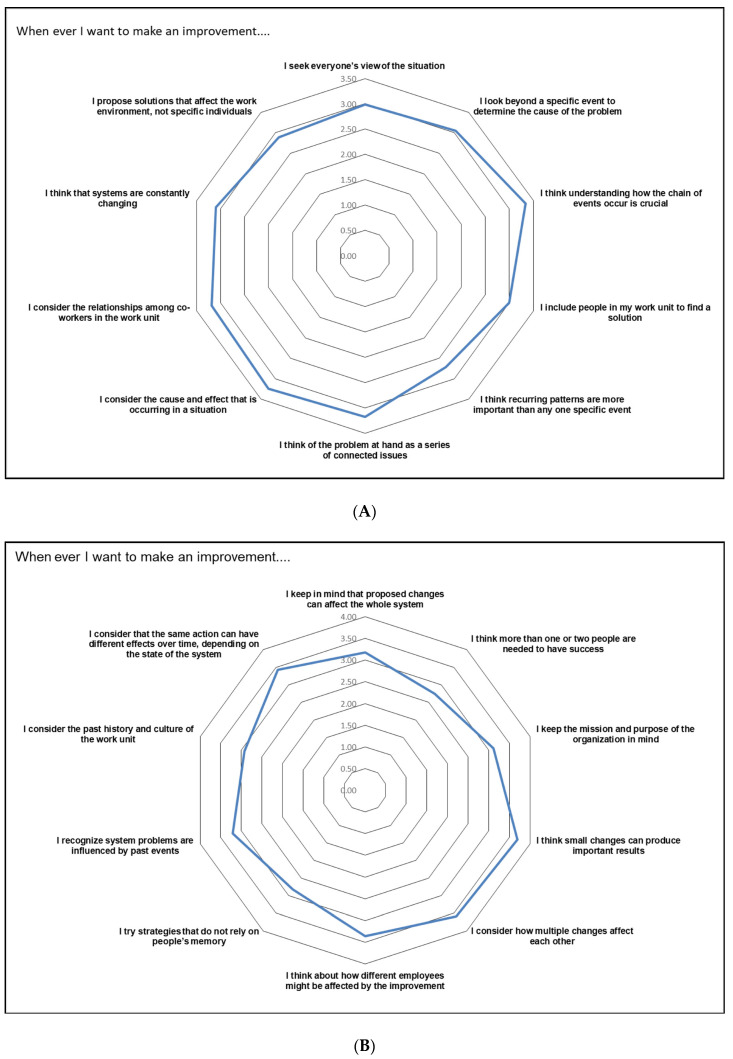
A graphical representation of the system thinking survey items with the most and least influential questions. (**A**), Part 1. (**B**), Part 2.

A radar chart was used to show the system thinking items that were the most apparent in the horror room setting. The following statements had the most agreement: “I think understanding how the chain of events occur is crucial”, “I think small changes can produce important results”, and “I consider how multiple changes affect each other”. The statements with the least agreement were “I think recurring patterns are more important than any one specific event” and “I think more than one or two people are needed to achieve success”. The radar chart of system thinking items is displayed in Figure 2A,B. 

**Figure 3:**
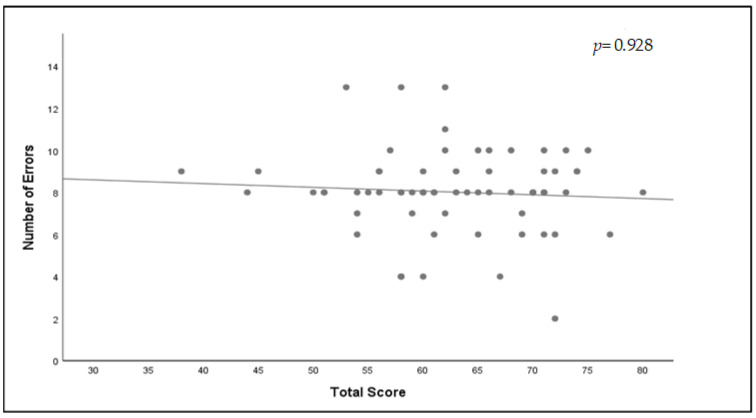
Correlation between the number of errors students detected and their system thinking score with correlation coefficient and *p*-value.

**Figure 4:**
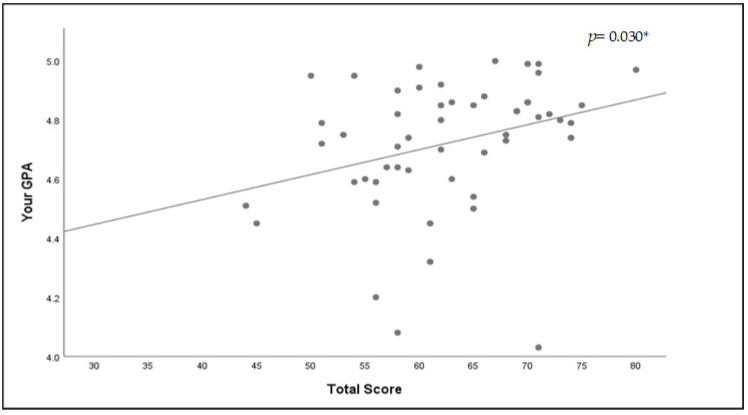
Correlation between GPA and system thinking score with correlation coefficient and *p*-value. Note. *p*-values < 0.05 were considered significant. * Spearman’s correlation coefficient = 0.27, *p* = 0.03.

Regarding the relationship between the number of errors identified and STS, no association was found (Spearman’s correlation coefficient = 0.01, *p* = 0.928; Figure 3). However, an association was found between GPA and STS (Spearman’s correlation coefficient = 0.27, *p* = 0.03; Figure 4).

## 4. Discussion

This work clearly demonstrates that the students were aware of the basic concepts of system thinking, as evidenced by their strong agreement with related statements. This study also reveals areas that need improvement, as the radar chart shows. Moreover, the study indicates that although the students were theoretically aware of system thinking concepts, they did not always reflect this knowledge in practice, as most students identified less than 50% of the errors present in the “horror room” setting. 

The College of Pharmacy students who participated in this study had a higher-than-average system thinking score [10]. This could be related to the didactic course they took, which included a 2-h lecture dedicated to system thinking. The students’ high scores suggest that didactic teaching is an effective methodology to teach system thinking. However, their practical application of system thinking knowledge and related concepts needs to be evaluated. 

The “horror room” setting can be utilized to teach root cause analysis and communicate the need for a culture of change as essential aspects of patient safety [18]. This recommendation is based on the students’ high agreement with related statements such as “I consider the cause and effect that is occurring in a situation”, and “I think understanding how the chain of events occur is crucial”, which represent essential determinants in root cause analysis. The relationship between system thinking and root cause analysis is known in digitalization [21]; however, this has rarely been explored in healthcare. 

No studies have linked system thinking to GPA. The students with the highest degree of agreement with some of the STS statements were those with a higher GPA, suggesting that GPA could be a marker of system thinking. The “horror room” setting is a valid tool that could help nurture system thinking based on the link with a higher GPA, and it should be used to enhance practical applications of system thinking, which the students in this research lacked. 

However, understanding system thinking concepts is insufficient to identify errors and solve problems within a team, as was evident in the activity, where students showed high STS but only identified 50% of the errors the activity design incorporated. Students showed positive responses regarding knowing the concept of system thinking. However, during the activity this knowledge was not translated into skills to identify the errors. This was evident by the fact that only 50% of the designed errors were identified by the students. Students need to practice and apply system thinking concepts in a simulated environment and learn how to think systematically by identifying the root of problems and proceeding with a systematic approach in the context of practice in different scenarios. Although the activity presented in this research is promising in terms of assessing system thinking, it needs to be applied on a larger scale involving an inter-professional team.

The practical implications of this research are tremendous. During write up, the lack of evidence on system thinking and “horror room” settings were barriers. No studies have explored system thinking from this perspective, so no comparison is available for elaboration. “Horror room” simulation settings can constitute a method for teaching and assessing students on various important concepts in healthcare education such as system thinking, teamwork, patient safety, and inter-professional education [14,18].Given the educational benefit, combining practice using simulation and didactic lecturing could be the best method to produce competent healthcare professionals. 

This study’s limitations include the use of a single site involving only women students, its cross-sectional design, and the small number of participants, which could impact generalizability.

This study has multiple strong points. To date, it is the first study to use the novel “horror room” setting to assess system thinking. Additionally, all extant studies examining system thinking have been in nursing, and the present study is the first in pharmacy. 

## 5. Conclusions

In conclusions, system thinking was found to be related to GPA but not to the number of errors students detected. This needs to be investigated further in larger multidisciplinary settings. The “horror room” is a feasible and reliable setting to teach and assess system thinking.

## Data Availability

Data is contained within the article and supplementary material. The datasets used and/or analyzed during the current study are available from the corresponding author on reasonable request.

## Supplementary Materials

The following supporting information can be downloaded at: https://www.mdpi.com/article/10.3390/healthcare11010066/s1, Supplementary Materials S1: Patient case, settings, activity and errors; Supplementary Materials S2: Reply for mail.
