# Supplementary material for: Assessing System Thinking in Senior Pharmacy Students Using the Innovative “Horror Room” Simulation Setting: A Cross-Sectional Survey of a Non-Technical Skill
Source: Healthcare (Basel). 2022 Dec 26;11(1):66. doi: 10.3390/healthcare11010066 (PMC9818965; doi:10.3390/healthcare11010066)
Supplement: Supplementary file 1 [file healthcare-11-00066-s001.zip › Supplementary Materials S1.pdf]

Supplementary Materials S1:

Patient case, settings, activity and errors

Case summary:

MS, a 68-year-old woman, was admitted to the hospital for the treatment of Streptococcus pneumoniae meningitis. On arrival, she was started on ceftriaxone 2g IV every 12 hours and improved during the next few days. On day 10 of antibiotic therapy, she developed cramping abdominal pain and diarrhea which was found to be Clostridium difficile infection, so she was started on Vancomycin 125 mg PO every 6 hours.

Patient characteristics:

A known case of Clostridium Difficile infection.

Co-morbid conditions:

GERD for 2 years on Pantoprazole.

HTN for 10 years on Lisinopril.

DM type 2 for 5 years on Insulin Glargine and Insulin Aspart.

Recurrent unprovoked DVT in 2016, on Warfarin.

Patients has Penicillin allergy.

Environment/setting/location:

Inpatient (Patient Room) at the Simulation Lab, College of Pharmacy, King Saud University.

Equipment, and supplies:

1- A hospital bed and a mannequin.
2- Hospital bedside table.
3- Curtains.
4- Sink for washing and a sanitizer.
5- Hospital IV drip stand and bag.
6- Medication record.
7- NPO sign.
8- Vital signs monitoring equipment with a display.
9- An unlabeled filled syringe on the bedside table

10- Insulin pens on the bedside table (Glargine or Aspart).
11- A plate of green salad on the bedside table.
12- Medication and syringes (unlabeled).
13- PICC line in the mannequin's arm with a wet and soiled dressing that is labelled with the date of last dressing change and insertion (two weeks back).
14- Empty glove container
15- Empty gown container

Working group task:

Students are handed the patient's file before entering the room. The file contains the patient's information, active problems, history, medications, orders, and the MAR summary.

The trainer will:

-Observe the student

-Document the number of error identified by student groups

Errors:

**Error classification**	**Error description**
**Medication related issues (5 Rs)**	1- ID bracelet with name not matching the name on the patient room.
	2- IV drip labelled "Ampicillin 2 gm in 0.9% NS- (1 mg/50 mL)" despite note of ampicillin allergy in the record.
	3- Drug is wrong concentration/Wrong medication / dose/dilution (vancomycin)
	4- Missed dose in the chart
	5- Pantoprazole (missed medication in medication chart).
	6- Instructions to given oral instead of IV despite NPO
	7- Intravenous (IV) drip site not dated
	8- Home medication should be not in the room
	9- Unlabeled pills and syringes in the room

	10- Improper storage(insulin not stored)
	11- Expired medication
	12- Insulin type is different than chart
	13- Warfarin and food interaction
**Hospital acquired infection**	1- Patient with inappropriate/unnecessary catheter / and exposed
	2- Empty alcohol-based hand sanitizer and soap dispensers in patient with presumed CDI
	3- Empty glove container
	4- Empty gown container
	5- Patient is lying flat, creating a risk for aspiration pneumonia
**Deep venous thrombosis**	1- No prophylaxis written in medication list for DVT
**Fall risk**	1- Side rails down or any precaution, even though patient is a 'fall risk'
	2- No fall risk sign/bracelet despite documentation in chart
**Nothing by mouth (NPO)**	1- Open food and drink should not be on tray table
	2- NPO

Objectives of The Activity:

The learner will be able to:

- Apply the system approach in analysing and preventing adverse events.
- Collaborate with other students while performing the task to engender a team approach to patient care.
- Communicate with other students while performing the task to engender a team approach to patient care.

- Identify situational and personal factors that are associated with the increased risk of errors.
- Gather information about patient risk
- Identify and report Adverse drug events through appropriate channels.
- Identify errors based on International Patient Safety Goals (IPSG):
    - Goal 1: Identify Patients correctly
    - Goal 2: Improve effective communication
    - Goal 3: Improve the safety of high alert medications
    - Goal 4: Ensure safe surgery
    - Goal 5:Reduce the risk of health care associated infections
    - Goal 6: Reduce the risk of patients harm resulting from falls
- Identify Medication safety problems through ensuring the five rights (patient, medication, dose, route, time).

Student Preparation (Pre-requisite knowledge/activities):

1. The session will be after the sixth lab.
2. Students need to incorporate their background knowledge and common sense in the context of:
    - Ensuring the five rights, including selecting the appropriate antibiotics based on patients' diagnosis.
    - Basic infection control measures.
    - Allergies, and drug-food interactions.
    - Indicating the high risk of fall patients.
    - Drug storing conditions.
