# Supplementary material for: Assessing System Thinking in Senior Pharmacy Students Using the Innovative “Horror Room” Simulation Setting: A Cross-Sectional Survey of a Non-Technical Skill
Source: Healthcare (Basel). 2022 Dec 26;11(1):66. doi: 10.3390/healthcare11010066 (PMC9818965; doi:10.3390/healthcare11010066)
Supplement: Supplementary file 1 [file Supplementary Materials S2.pdf › Supplementary Materials S2.pdf]

From: **Mary Dolansky** mad15@case.edu
Subject: Re: System Thinking scale Permission to use
Date: 26 April 2021 at 4:42 am
To: Lubna A. Aljaffali laljaffali@ksu.edu.sa

MD

Hello,

So very sorry for the delay in responding.

Yes, you have our permission to use the systems thinking scale. Will you be translating it?

Thank you for sharing any results or feedback with us.

Kind regards,

Mary

On Thu, Mar 11, 2021 at 4:09 AM Lubna A. Aljaffali <laljaffali@ksu.edu.sa> wrote:

> Dear Dr Mary
>
> I hope you are doing fine. I am a faculty in the collage of pharmacy in King Saud University, Riyadh ,Saudi Arabia. We introduced the patient safety course to our seniors that is adopted from the WHO curriculum guide for patient safety,one of the important topics in this curriculum is the system approach . We are trying different methods to assess our students system thinking skills and how they engage in problems solving as it is an important competence needed when they graduate. I found your study and it similar to what we are seeking to explore for assessing our students and to do further research on. I would like to ask your permission to use it, the survey will be cited as your work in any publication.
>
> Thank you
>
> Best regards,
> Lobna
>
> **Lobna Al Juffali, Msc, PhD**
> Head of the Pharmacy Education Unit
> Assistant Professor
> Clinical Pharmacy Department
> College of Pharmacy
> King Saud University
> Mobile:00966505172290
> P.O. Box 26572 Riyadh 11496
> Kingdom of Saudi Arabia
>
> ---
>
> Disclaimer:
> This communication is intended for the above named person and is confidential and / or legally privileged. Any opinion(s) expressed in this communication are not necessarily those of KSU (King Saud University). If it has come to you in error you must take no action based upon it, nor must you print it, copy it, forward it, or show it to anyone. Please delete and destroy the e-mail and any attachments and inform the sender immediately. Thank you.
> KSU is not responsible for the political, religious, racial or partisan opinion in any correspondence conducted by its domain users. Therefore, any such opinion expressed, whether explicitly or implicitly, in any said correspondence is not to be interpreted as that of KSU.
> KSU may monitor all incoming and outgoing e-mails in line with KSU business practice. Although KSU has taken steps to ensure that e-mails and attachments are free from any virus, we advise that, in keeping with best business practice, the recipient must ensure they are actually virus free.
